# Upstream and Downstream Cardiovascular Changes in Rheumatic Mitral Stenosis: An Update

**DOI:** 10.3390/jcm14082639

**Published:** 2025-04-11

**Authors:** Estu Rudiktyo, Arco J. Teske, Emir Yonas, Ade M. Ambari, Maarten J. Cramer, Marco Guglielmo, Tommaso Semino, Bambang Budi Siswanto, Pieter A. Doevendans, Amiliana M. Soesanto

**Affiliations:** 1 Department of Cardiology and Vascular Medicine, Faculty of Medicine, Universitas Indonesia—National Cardiovascular Center Harapan Kita, Jakarta 11420, Indonesia; esturudi@gmail.com (E.R.); e_yonas@windowslive.com (E.Y.); dr_ade_meidian@yahoo.co.id (A.M.A.); bambbs@gmail.com (B.B.S.); amiliana14@gmail.com (A.M.S.); 2 Department of Cardiology, Division Heart and Lungs, University Medical Center Utrecht, 3584 Utrecht, The Netherlands; a.j.teske-2@umcutrecht.nl (A.J.T.); m.j.m.cramer@umcutrecht.nl (M.J.C.); p.doevendans@umcutrecht.nl (P.A.D.); 3 Chair of Cardiovascular Disease, Department of Internal Medicine and Specialties (Di.M.I.), University of Genoa, 16126 Genova, Italy; tommy.semino@gmail.com; 4 Central Military Hospital, 3584 Utrecht, The Netherlands; 5 Netherlands Heart Institute, 3511 Utrecht, The Netherlands

**Keywords:** rheumatic heart disease, mitral stenosis, ventricular dysfunction, atrial fibrillation, pulmonary hypertension

## Abstract

Rheumatic heart disease (RHD) and its complications are major health problems worldwide, especially in developing countries, owing to their high prevalence. Mitral stenosis (MS) is one of the most common lesions in RHD, either isolated or in combination with mitral regurgitation, and eventually leads to atrial fibrillation (AF), congestive heart failure, pulmonary hypertension (PH), and other complications, including ischemic stroke or limb ischemia, if not promptly diagnosed and treated. Recent studies have suggested that MS affects the cardiovascular system beyond mere obstructions. The presence of MS in RHD causes significant changes in the cardiovascular system, both upstream and downstream, affecting both the left and right ventricles. Rheumatic MS causes significant structural changes through inflammatory pathways and hemodynamic changes, owing to its obstructive effects. This review aims to discuss the vast changes in the cardiovascular system caused by rheumatic MS.

## 1. Introduction

Rheumatic heart disease (RHD) and its complications are major health problems worldwide, especially in developing countries, due to their high prevalence. Data published by The Global Burden of Disease showed that at least 40.5 million people suffered from RHD worldwide in 2019 [1]. From a broader viewpoint, this number markedly surpasses the global tuberculosis patients count of 2018, encompassing roughly 10 million individuals [2]. Mitral stenosis (MS) is one of the most common lesions in RHD, either isolated or in combination with mitral regurgitation, and eventually leads to atrial fibrillation (AF), congestive heart failure, pulmonary hypertension (PH), and other complications, including ischemic stroke or limb ischemia, if not promptly diagnosed and treated [3,4,5].

**Figure 1:**
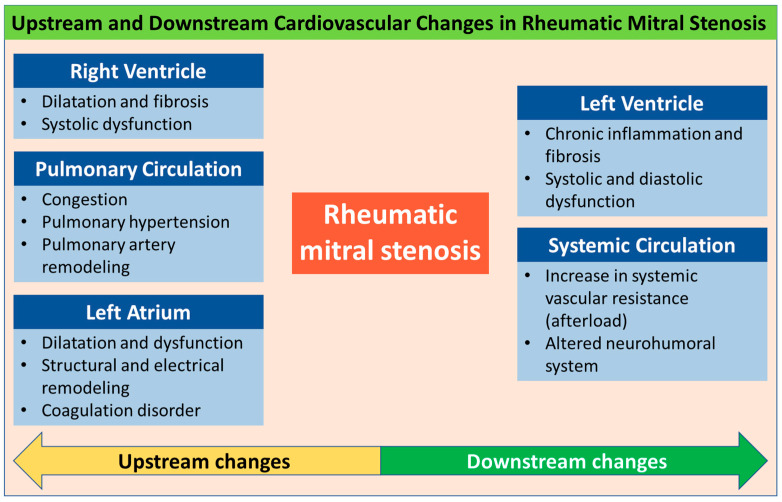
Central illustration: cardiovascular changes in patients with rheumatic mitral stenosis.

The recent literature suggests that the cardiovascular impact of MS in RHD goes beyond mere flow obstruction resulting in elevated pressures upstream of the valve and also encompasses atrial remodeling and fibrosis [6,7], left ventricular (LV) interstitial fibrosis, and dysfunction [8]. A thorough understanding of these cardiovascular changes and adaptations in patients with rheumatic MS is essential for clinical decision-making and planning personalized interventions. This article reviews upstream and downstream changes in the cardiovascular system in rheumatic MS, including systemic and pulmonary circulation (Figure 1).

## 2. Methods

This manuscript presents a narrative review to provide a comprehensive overview of the topic, including an extensive discussion of the alterations in cardiac and circulatory function associated with rheumatic mitral stenosis. Furthermore, it focuses on novel imaging modalities, such as cardiac magnetic resonance imaging and advanced echocardiographic techniques, specifically global longitudinal strain and myocardial work assessment. This article aims to provide clinicians with a thorough review of MS, which is a significant global health concern.

## 3. Changes in Mitral Valve

**Figure 2:**
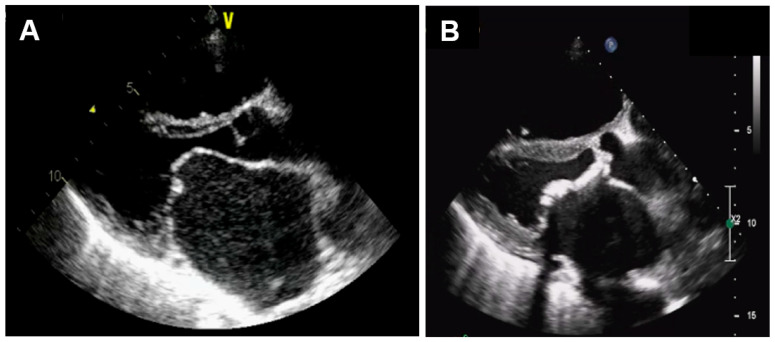
Echocardiographic findings of mitral valve in rheumatic mitral stenosis. (**A**) Parasternal long-axis view of patient in earlier stage, showing normal thickness in valve with minimal calcification. (**B**) Parasternal long-axis view of different patient with concomitant rheumatic aortic stenosis presenting in later stage, showing extensive calcification of anterior mitral leaflet and aortic valve.

In the initial stage of rheumatic fever, small verrucous-like nodules emerge due to the formation of blood clots along the heart valve closure lines [9]. These growths do not cause any damage to the valve leaflets, and valve function remains mostly normal. Chronic inflammation stemming from single or multiple occurrences of rheumatic fever may cause valve malfunction in individuals who are genetically susceptible and left untreated [9]. As a general pathological finding, thick and stiff mitral valve specimens from patients with end-stage disease are due to extensive fibrosis. Mutagaywa et al. demonstrated from the histopathological evaluation of mitral valve specimens in MS patients that the presence of specimens showed leukocytic infiltrates, fibrinoid degeneration, Aschoff bodies, calcified lesions, and fibrosis [10]. While the leaflets are usually less fibrotic and flexible in most patients younger than 30 years of age, they are scarred and rigid in older patients [9]. Long-standing inflammation can result in the formation of fibrinous vegetations on the anterior mitral leaflet and scarring of the leaflets, ultimately leading to valvular stenosis. In this state, the valve becomes immobile and cannot be fully opened. Figure 2 shows echocardiographic findings from patients in the early (A) and late (B) stages of rheumatic MS.

## 4. Upstream Changes

### 4.1. Left Atrium

#### 4.1.1. Structural Remodeling

Left atrial (LA) remodeling is the response of cells including cardiomyocytes in the atrium to various stressors, including electrical, mechanical, and metabolic ones [11]. Remodeling is initially an adaptive mechanism. Nevertheless, when it takes place as a result of a persistent pathological stimulus, it can be considered abnormal and has been linked to an elevated likelihood of adverse cardiovascular events. In patients with valvular heart disease, including rheumatic MS, the magnitude of LA structural or functional alteration varies and depends on the type, duration, and severity of valve lesions [12,13]. LA remodeling appears to result in metabolic changes with a shift in the energy source to fetal glycolysis via the beta-oxidation of fatty acids, resulting in a decrease in energy production [14]. Also, increases in atrial and brain natriuretic peptides (BNPs), angiotensin II, transforming growth factor beta, and aldosterone that promote atrial fibrosis have been reported [12].

**Figure 3:**
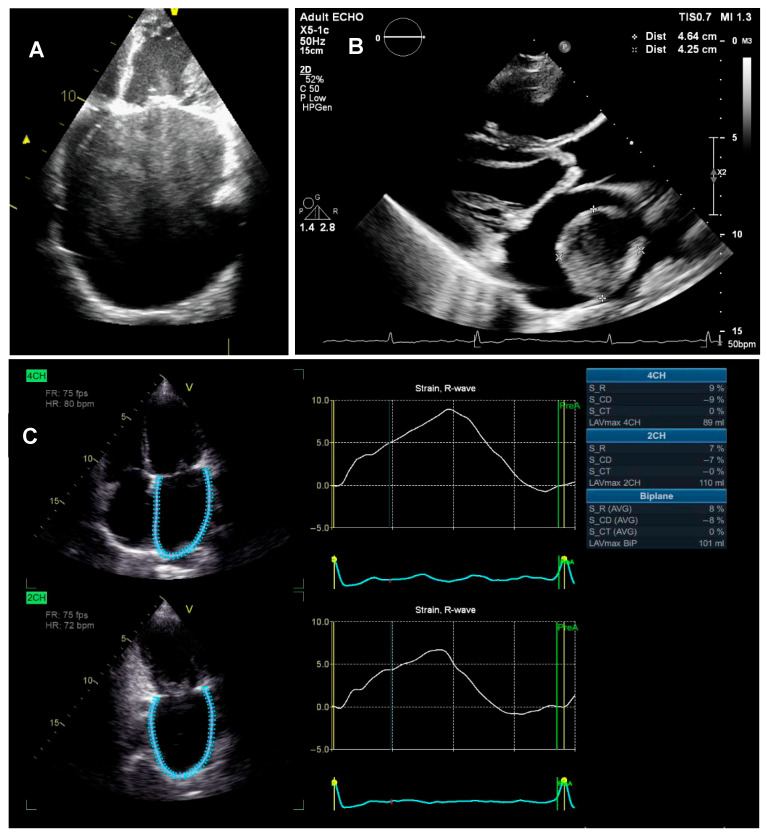
Remodeling of left atrium. (**A**) Markedly dilated left atrium with dense spontaneous echo contrast. (**B**) Large left atrial thrombus. (**C**) Two-dimensional speckle-tracking strain demonstrating impaired left atrial function in conduit, contractile, and reservoir phases.

In patients with MS, a combination of elevated LA pressure and inflammation in the atrium secondary to the rheumatic carditis is accompanied by the progressive interstitial fibrosis of the atrial wall with the disorganization of the atrial muscle, LA dysfunction, and the subsequent dilatation of the LA (Figure 3A) [11]. A study using delayed enhanced cardiovascular magnetic resonance (CMR) reported that atrial fibrosis was found in 66.6% of patients with rheumatic MS and related to the presence of AF [7]. Elkareem et al. reported that there was a significant negative correlation between the mitral valve area (MVA) and LA volume index (LAVi) [11]. Nonetheless, this finding conflicted with what was reported by the study of Iqbal et al., which showed no significant correlation between MVA and LA size [15]. Rudiktyo et al. reported that in significant isolated rheumatic MS (MVA less than 1.5 cm^2^), LA enlargement was found in 90.8% of patients with a mean LAVi of 110 ± 90 mL/m^2^ [3]. The measurement of LA size is important, due to a significant correlation between the dilatation of the LA and an increased risk of AF and ischemic stroke [16].

#### 4.1.2. Electrical Remodeling

Atrial tachyarrhythmias including atrial flutter and AF are caused by triggers that generate ectopic activity or changes in the substrate that promote reentry [13]. The latter have an effect through a pathophysiological pathway, which involves a shortened action potential duration, a reduction in refractory periods, or changes in atrial contractility mediated by an alteration in calcium influx and potassium efflux from the cardiomyocytes [12]. Electrical remodeling promotes a re-entry-prone environment created by alterations in ion channels, pumps, and exchangers with the abnormal distribution and expression of gap junction connexin hemichannels that electrically link the cardiomyocytes [17]. The presence of atrial fibrosis also promotes changes in cellular coupling and impulse propagation, which can serve as a potential cause for initiating and perpetuating re-entrant atrial arrhythmias [12].

John et al. demonstrated that in MS, there is a reduction in conduction velocity and an increase in effective refractory periods in both atria, as well as a decrease in atrial voltage and an increase in electrical scar formation [18]. However, it remains unclear whether electrical remodeling occurs before or after structural remodeling [18]. Regardless, the presence of both types of remodeling has been associated with increased AF inducibility. Atrial stretch resulting from pressure overload is another mechanism contributing to AF. Fan et al. reported that assessments performed before and after percutaneous transvenous mitral commissurotomy (PTMC) showed that LA pressure is correlated with AF inducibility [19]. These findings suggest that some forms of electrical remodeling may be related to pressure overload-induced atrial stretch, which could potentially be reversed if addressed before extensive structural remodeling occurs.

#### 4.1.3. Coagulation Disorder

Left atrial thrombus, with the inherent risk of causing systemic embolization including embolic stroke, is a catastrophic and common complication of MS. Although the pathogenesis of LA thrombus is incompletely understood, Virchow’s triad has been identified as a possible mechanism of thrombus formation [20]. Yamamoto et al. reported that coagulation activity is significantly elevated in the LA of patients with MS even during the administration of anticoagulant agents, which might contribute to the pathogenesis of thromboembolism [21]. They also found that platelet activity is not significantly increased in the LA of these patients. Peverill et al. reported that increased regional LA coagulation activity in MS occurs in the presence of LA spontaneous echo contrast, both in sinus rhythm and AF, and is associated with normal systemic coagulation activity [22]. Figure 3B shows the presence of large LA thrombus in a patient with rheumatic MS.

#### 4.1.4. LA Dysfunction

There are three components of LA function: First, the LA serves as a reservoir during left ventricular systole, enabling blood to be gathered near the closed mitral valve. Second, the LA undergoes passive shortening during early LV diastole, in response to early LV filling (conduit phase). Finally, it functions as a booster pump during late diastole after the P-wave due to atrial contraction [23]. Changes in LA function and size have been linked to unfavorable cardiovascular events.

A prominent sign of LA structural remodeling is dilatation. This is usually followed by a change in LA function, due to an increase in interstitial fibrosis. Atrial booster pump and reservoir dysfunction are compensated by increased conduit function [12]. Measured with various parameters, decreased LA function was found in MS patients. A study by Amshala showed that E’ and A’ septal tissue Doppler velocities were observed in patients with MS. Patients with MS had lower velocities in the lateral wall of the LA than healthy controls [24]. After mitral balloon valvuloplasty, the lateral E’ velocity increased (*p* < 0.001). Furthermore, the MS patient group showed lower LA strain at ventricular end-systole than the controls [24]. Figure 3C shows examples of measurements of LA function using two-dimensional speckle-tracking strain in a rheumatic MS patient.

### 4.2. Pulmonary Circulation

Severe MS leads to elevated LA pressure, which, in turn, increases pulmonary venous and capillary pressures, resulting in pulmonary congestion. It also causes pulmonary arteriolar vasoconstriction, obliterative changes in the pulmonary vascular bed, and the development of pulmonary arterial hypertension [25]. Pulmonary hypertension is a common complication of MS. Rudiktyo et al. reported that increased PA pressure was observed in 49.3% of patients with isolated significant (moderate and severe) MS, significantly higher than the proportion of increased PA pressure in patients with isolated rheumatic mitral regurgitation (35.1%) or isolated rheumatic aortic regurgitation (5.9%) [3].

The primary mechanism by which elevated pulmonary arterial pressure (PAP) occurs in patients with left heart disease, including MS, is the backward transmission of high LA pressure (post-capillary PH). This can be estimated by measuring the pulmonary arterial wedge pressure using right heart catheterization. Although the transmitral gradient is a strong determinant of PAP, it is not the only factor [26]. Low atrioventricular compliance, which is a composite measure of LA and LV compliance, can predict systolic PAP independently of MVA and the mean diastolic pressure gradient [26]. Additionally, depending on the severity and duration of LA pressure elevation, the pathophysiology of PH in MS entails the structural remodeling of the pulmonary vascular bed (combined pre- and post-capillary PH), which is mediated by the vasoconstrictor endothelin-1 [27]. It has been demonstrated that the levels of endothelin-1 are three times higher in individuals with severe MS as compared to normal controls [27].

The presence of elevated PAP or PH in patients with mitral valve disease plays a pivotal role in decision-making for percutaneous or surgical intervention in the most recent valvular guidelines [28,29]. The current European Society of Cardiology (ESC) guidelines denote the role of PH as a marker of more advanced cases and give a IIa recommendation for PTMC in patients with severe MS (mitral valve area less than 1.5 cm^2^) with no or few symptoms but a systolic PAP > 50 mmHg [29]. Without the relief of mitral valve obstruction, observational data showed that the average survival was less than 3 years when significant PH occurred in patients with MS [30]. Unfortunately, in patients with severe PH with the remodeling of the pulmonary capillary bed, percutaneous or surgical interventions for the correction of valve lesions may have persistently elevated PAP, which was associated with adverse prognosis [31].

### 4.3. Right Ventricle

In MS, involvement of the right ventricle (RV) can arise from increased afterload secondary to the presence of PH. Additionally, it may be caused by an intrinsic myocardial process in the RV. Pande et al. demonstrated the presence of apoptosis in the RV of patients with rheumatic MS that occurred early even with low RV systolic pressure [32]. Rarely, rheumatic tricuspid valve lesions (regurgitation, stenosis, or both) may also cause a deleterious impact on the RV. The most frequently observed changes in the RV are dilation and reduced systolic function. According to a study conducted by Rudiktyo et al., the prevalence of RV dysfunction (as indicated by tricuspid annular plane systolic excursion or TAPSE less than 17 mm) among patients with isolated moderate or severe rheumatic MS was 46.1% [3]. This proportion was notably higher than that observed in patients with isolated rheumatic mitral regurgitation or isolated aortic valve abnormalities (24.6% and 4.0–13.4%, respectively) [3].

#### 4.3.1. Structural Changes

The involvement of the RV myocardium in rheumatic processes is less well established and studied than that of the LV myocardium, which will be discussed later. Pande et al. reported evidence of apoptosis, fibrosis, and fat deposition in the RV myocardium related to the rheumatic process, independent of the pulmonary hypertension status [33]. They demonstrated an increase in the expression of pro-apoptotic genes (cytochrome c, caspase 3, Bax, and Fas) and anti-apoptotic genes (Bcl-2) in the RV of patients with RHD [32,33]. However, no studies have shown whether this process is related to remodeling, dilatation, or RV dysfunction. Rudiktyo et al. showed that rheumatic carditis was not significantly associated with decreased RV contractility in patients with rheumatic mitral regurgitation [34]. This finding can be partly explained by the fact that right-sided primary rheumatic valve lesions are less common than left-sided valve lesions in RHD patients [3]. Therefore, it is proposed that the direct involvement of the RV myocardium in the inflammatory process due to rheumatic carditis is less significant than that of the LV myocardium.

#### 4.3.2. Functional Changes

Theoretically, the mechanisms of reduced RV function in RHD can be categorized into two types: (a) decreased RV function due to increased RV afterload or PH and (b) intrinsic processes in the RV myocardium not caused by PH. Almost all conditions affecting the left heart can impact the RV, including mitral and aortic valve disease. Both stenosis and regurgitation in the left-sided valves, especially the mitral valve, increase the LA pressure, resulting in an increase in backward pressure to the pulmonary veins. This pressure increase is then transmitted to the pulmonary capillary bed, leading to complex processes that result in PH, which is associated with poor prognosis. If left untreated for a long period, PH eventually causes RV dilation and reduced function. RV dilation also leads to the dilation of the tricuspid valve annulus, causing tricuspid regurgitation, further exacerbating dilation, and reducing RV contractility [35]. However, as mentioned previously, no study has demonstrated a relationship between this intrinsic myocardial process and decreased RV function. The connection between RV dysfunction and an increased risk of both postoperative and long-term morbidity and mortality in patients who underwent mitral or mitral–aortic valve surgery including RHD cases was established in several studies [36,37].

## 5. Downstream Changes

### 5.1. Left Ventricle

#### 5.1.1. Structural Alterations

**Figure 4:**
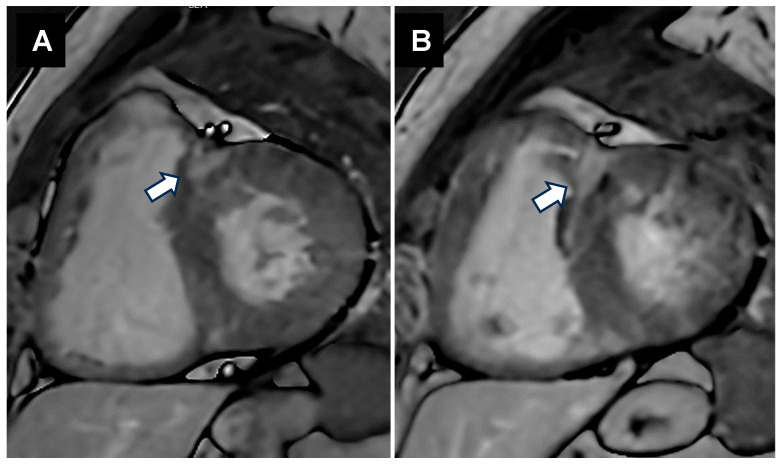
Cardiovascular magnetic resonance images of patients with rheumatic mitral stenosis during diastole (**A**) and systole (**B**). White arrows indicate regions of late gadolinium enhancement (LGE).

In acute rheumatic fever (ARF), an inflammatory process occurs not only in the valves but also in the myocardial and pericardial layers. This inflammatory process, often referred to as pancarditis, serves as a precursor to rheumatic heart disease (RHD) [38,39]. Left ventricle myocardial involvement was confirmed by the finding of Aschoff bodies in the myocardium [40,41]. These nodules were pathognomonic findings in RHD and were also found in cardiac valve tissue [41]. Studies using CMR with delayed gadolinium enhancement demonstrated LV fibrosis in 91.5% of patients with rheumatic MS (Figure 4) who did not have coronary heart disease [42] and only 18.2% of patients with chronic rheumatic mitral regurgitation [6]. Chronic inflammation in patients with ARF and RHD is associated with the development of focal myocardial fibrosis. During ARF, pancarditis is the dominant cardiac manifestation, whereas valve scarring and subsequent lesions are most apparent in the chronic stage. Pathological studies have demonstrated that fibroid necrosis in the interstitial tissue of the myocardium, followed by the granulomatous phase and the subsequent presentation of Aschoff bodies, may play a role in causing disarray within the myocardium [43].

However, there is conflicting evidence regarding whether ARF directly causes myocarditis. Several studies have reported that myocarditis is not characteristic of rheumatic carditis. Oran et al. showed that myoglobin, creatine kinase, and troponin-I levels in patients with ARF with carditis with or without cardiomegaly remained normal during a 3-week follow-up, showing an absence of significant myocardial damage [44]. Kamblock et al. measured cardiac troponin-I levels in 95 ARF patients. They concluded that there were no cardiac troponin-I elevations, suggesting no significant myocardial involvement during acute episodes [45]. Furthermore, Narula et al. used myocardial biopsies in 89 patients with ARF and RHD to identify active myocarditis [46]. They suggested that the examination of myocardial biopsy during ARF does not contribute to clinically evident myocarditis, as the extent of myocardial damage is limited.

#### 5.1.2. Changes in Systolic Function

Obstructed flow from the LA leads to LV underfilling. Patients with MS have been reported to have elevated LV filling pressure, greater LV end-diastolic volume, reduced contractility, and decreased calculated LV compliance. However, these abnormalities are typically observed infrequently and only slightly exceed the normal limits. Nevertheless, a decrease in LV compliance significantly correlates with the degree of PH, RV filling pressure elevation, the ratio of the filling pressures of both ventricles, and LV end-systolic eccentricity [47].

Left ventricular systolic function is one of the most important prognostic markers in patients with cardiovascular diseases, including in patients with valvular heart disease. Previously, it was reported that patients with rheumatic MS often display LV systolic dysfunction, and the rate of reduced LV ejection fraction (EF) may be as high as 33% [48,49,50,51]. Hemodynamic and myocardial factors have been linked to left ventricular (LV) dysfunction, including decreased LV filling, the chronic inflammation of the heart muscle, the scarring of the sub-valvular apparatus, reduced LV compliance and diastolic dysfunction, increased afterload, abnormal interaction between the right and left heart chambers (ventricular interdependence), and PH [52,53,54]. The presence of myocardial fibrosis in the LV was also reported to be associated with decreased EF and global longitudinal strain (GLS) in patients with rheumatic MS [55,56].

Sengupta et al. reported that mitral annular velocities, measured using tissue Doppler imaging (TDI), but not EF, increased after PTMC. The improvement in velocities correlated with the changes in the MVA [57]. An electron microscopy investigation in MS patients demonstrated that ultrastructural changes such as myocyte injury and loss, the disproportion of the mitochondria-to-myofibril ratio, and severe myofibrillolysis occurred in myocardial cells regardless of LV function, and these changes were not associated with the MVA [58]. Also, patients with LV dysfunction showed more myofibrils loss. Bilen et al. reported that patients with MS had lower LV GLS values when compared with normal controls [59]. Interestingly, there were similarities in GLS values between the mild, moderate, and severe MS groups [59]. These studies indicate that subclinical LV dysfunction is influenced by myocardial factors rather than hemodynamic factors. Additional research to verify the potential for anti-inflammatory medications to reverse myocardial fibrosis is currently being conducted [60].

**Figure 5:**
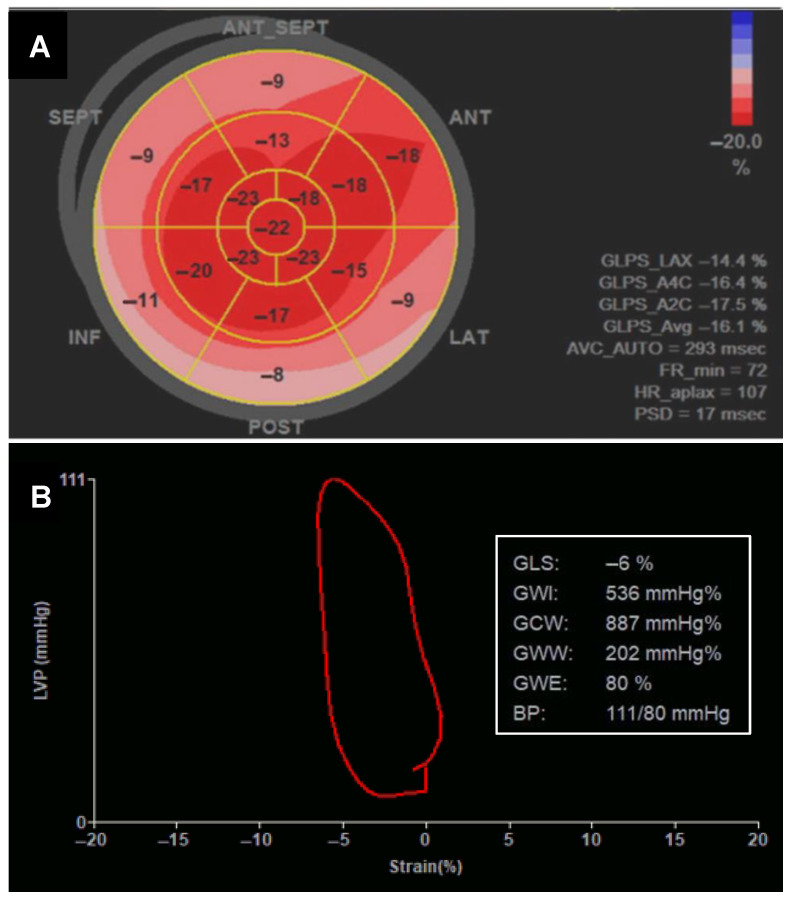
Global longitudinal strain and global LV myocardial work in patients with severe rheumatic mitral stenosis. (**A**) Bull’s eye of left ventricular global longitudinal strain, showing reduced value (−16.1%). (**B**) LV pressure strain-loop curve from different patients and corresponding myocardial work parameters, showing severely reduced global work efficiency.

High afterload has also been hypothesized to be an additional possible cause of reduced LV systolic function in patients with MS. Gash et al. proposed that a reduction in EF may be attributed to an unmet high afterload, despite an increase in preload resulting from the decrease in the mitral valve area in MS. They proposed that high afterload results from inadequate end-systolic wall thickness, which increases wall stress at a normal LV systolic pressure [51]. Wisenbaugh et al. studied the effect of PTMC on afterload, reporting no significant decrease despite a significant increase in preload [61]. A contemporary study by Rudiktyo et al. using noninvasively measured myocardial work (MW) observed the presence of subtle LV dysfunction in patients with severe MS as demonstrated by significantly reduced LV GLS compared to healthy controls despite similar EF. Also, the inefficiency of work performed by the LV as shown by significantly higher wasted work and lower constructive work compared to in normal individuals was found [8]. However, a study conducted by Kamblock reported that there was no decrease in EF in patients with ARF. This study concluded that there was no significant myocardial injury in ARF, although Aschoff nodules were found in the myocardium [45]. These findings differ from the previously established assumption that myocarditis also occurs in ARF. However, Kamblock et al. measured LV systolic function using EF, which could not detect subtle LV dysfunction. Contemporary echocardiographic parameters such as GLS and MW have been reported to detect subtle or subclinical LV dysfunction [8]. Figure 5 shows examples of measurements of LV GLS and MW in rheumatic MS patients.

#### 5.1.3. Diastolic Dysfunction in MS

Directly related to the severity of MV obstruction, LA pressure is elevated in patients with significant MS due to the inability of the obstructed valve to facilitate adequate passive atrial emptying during the diastolic phase, hence relying heavily on the atrial kick in patients with sinus rhythm. Consequently, the LV end-diastolic volume is significantly reduced, which in turn causes a low stroke volume. LV expansibility is impaired due to a rigid, thickened mitral valve complex and its attachment to the LV, leading to abnormal diastolic function [50]. In patients with concomitant LV diastolic dysfunction, symptoms of dyspnea exceed the severity of MS, and such patients may have persistent symptoms even after PTMC or valve surgery [62]. In normal conditions, negative LV intraventricular pressure generated in the early diastolic phase (diastolic suction) leads to low dependency on active LV filling (atrial contraction) during the diastolic phase. Sabbah et al. reported that this mechanism was lost in MS patients with diastolic dysfunction [63]. Eleid et al. showed that over 30% of subjects with MS have high LV end-diastolic pressure (LVEDP) based on invasive measurement [64]. Persistent symptoms and a higher incidence of additional procedures were observed in patients with elevated LVEDP when compared to those without significant diastolic dysfunction [64].

Concomitant conditions, such as coronary artery disease, systemic hypertension, diabetes mellitus, and aging, may contribute to abnormal diastolic function, adding to the difficulty of assessment. Echocardiographic measurements of diastolic function in patients with MS are different and more challenging than those in patients without significant mitral valve disease [65]. Furthermore, it is difficult to noninvasively determine whether elevated LA pressure is caused by the stenotic mitral valve, increased LVEDP, or both. Hence, the assessment of diastolic function in the population with MS is still not routinely performed despite its abovementioned importance. In patients with symptoms of dyspnea, these can exceed the severity of those of MS, and such patients may have persistent symptoms even after PTMC or valve surgery. Therefore, thorough diastolic function assessment should be performed in MS patients who exhibit unambiguous symptoms or have coexisting conditions that are typically associated with diastolic dysfunction [62,64]. Brain natriuretic peptides are linked with functional capacity and MVA in MS patients and may be useful in the evaluation of patients with equivocal symptoms [66].

### 5.2. Systemic Circulation

The flow-limiting obstruction of the mitral valve can lead to the underfilling of the LV, which can result in a lower stroke volume. To preserve cardiac output and peripheral perfusion, there may be an increase in heart rate and systemic vascular resistance (afterload). Initially, the relationship between increased afterload and reduced preload was believed to be inversely proportional. Nevertheless, subsequent studies have observed the presence of high afterload that remains uncompensated for by a reduction in preload [51]. It was hypothesized that higher afterload results from inadequate end-systolic wall thickness, which increases wall stress at a normal LV systolic pressure. Similar evidence of increased afterload was found by Kaku et al. and Mohan et al. in this patient population [49,67]. Another study demonstrated that successful valvuloplasty was not associated with significant decrease in afterload despite an apparent increase in preload [61]. Wisenbaugh et al. suggest that there may be other unmeasured factors, such as endothelin, that result in higher peripheral vascular resistance and vasoconstriction in this patient population [61]. According to Rudiktyo et al., a reduction in MW efficiency was observed in individuals with severe, isolated MS and preserved EF. This decrease in efficiency may be attributed to an increase in afterload [8], as MW also included afterload in the measurement, in addition to preload. Furthermore, there is evidence of increased neurohormonal activation in the systemic circulation [68].

## 6. Clinical Implications in Management Strategies

Understanding the alterations in the cardiovascular system in rheumatic MS is fundamental for comprehensive treatment, which comprises medical, surgical, or non-surgical interventions in selected cases. Medical management constitutes the cornerstone of initial treatment, encompassing diuretics to mitigate pulmonary congestion, digoxin, beta-blockers, or calcium channel blockers for heart rate control, and anticoagulation therapy to prevent thromboembolic events [69]. Penicillin prophylaxis is essential for preventing recurrent rheumatic fever and further valve deterioration [70]. These management strategies are primarily directed toward alleviating or preventing complications arising from pathological changes in the cardiac and circulatory systems.

Interventional procedures are often necessary in cases of more severe or symptomatic patients. The preferred initial interventional treatment for appropriate candidates is PTMC [69]. This minimally invasive technique involves the use of a balloon to expand the stenotic mitral valve, thereby increasing its area. This procedure has shown excellent outcomes in both the short and long term, significantly improving symptoms and hemodynamics. Surgical intervention remains a crucial option for patients unsuitable for PTMC or those with additional valve lesions. When possible, mitral valve repair is favored over replacement, owing to better long-term results and the avoidance of anticoagulation therapy [71]. However, the extent of valve damage often necessitates replacement. The durability of the repaired mitral valve is also problematic because inflammation from recurrent rheumatic fever episodes may cause new lesions in the repaired valve [72]. Recent surgical advancements include minimally invasive and robot-assisted approaches, potentially leading to faster recovery and reduced surgical trauma [73]. However, implementing such high-cost treatments may not be feasible in developing countries, where rheumatic MS cases are prevalent.

The current ESC/EACTS and ACC/AHA guidelines for managing valvular heart disease advocate for intervention (PTMC or surgery) in cases of clinically significant rheumatic MS (valve area ≤ 1.5 cm^2^) [28,68,74]. However, these guidelines lack specific recommendations for patients with reduced ventricular contractility or pulmonary hypertension. These conditions, which are particularly prevalent in developing nations, are associated with unfavorable outcomes and elevated treatment costs [26]. It is imperative to stress the importance of timely corrective procedures, whether percutaneous or surgical, before the onset of ventricular dysfunction or pulmonary hypertension. Additionally, the guidelines overlook the necessity of a specific post-procedural monitoring program, both in the short and long term. This oversight persists despite the established understanding that RHD-related inflammatory processes continue without proper prophylaxis, potentially affecting previously unaffected valves, compromising the longevity of repaired valves, and possibly leading to the deterioration of LV function over time [70].

## 7. Conclusions

Rheumatic mitral stenosis can cause significant changes in all cardiac chambers, including the systemic and pulmonary circulation. Advanced echocardiographic techniques and state-of-the-art cardiac imaging modalities facilitate the detection of subtle changes that may have previously gone unnoticed using conventional diagnostic methods. These alterations may include, but are not limited to, impaired left and right ventricular function, pulmonary hypertension, increased systemic afterload, and atrial remodeling, which can precipitate arrhythmia and increase the risk of thromboembolic events. The progression of these changes is correlated with delayed patient presentation, often resulting in more severe manifestations and poorer outcomes. Timely diagnosis, a comprehensive understanding of these pathological changes, and appropriate definitive management are crucial to prevent long-term adverse effects on the cardiovascular system.

### Future Directions

While recognizing the importance of understanding cardiovascular changes in rheumatic MS, future investigations should focus on elucidating the underlying pathophysiological mechanisms driving these changes. This includes the identification and validation of novel biomarkers that can predict disease progression and response to therapy. Moreover, research should aim to translate these findings into clinically applicable tools for early detection and targeted interventions.
